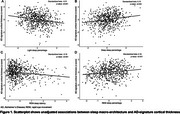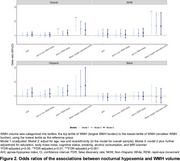# Sleep macro‐architecture, nocturnal hypoxemia, and Alzheimer’s Disease‐related MRI patterns among diverse older adults

**DOI:** 10.1002/alz70862_110235

**Published:** 2025-12-23

**Authors:** Dillys Xiaodi Liu, Meredith N. Braskie, Clémence Cavaillès, Carrie B. Peltz, Susan Redline, Kristine Yaffe

**Affiliations:** ^1^ Department of Psychiatry and Behavioral Sciences, University of California, San Francisco, San Francisco, CA USA; ^2^ Imaging Genetics Center, Mark and Mary Stevens Neuroimaging and Informatics Institute, Keck School of Medicine, University of Southern California, Marina del Rey, CA USA; ^3^ NCIRE‐The Veterans Health Research Institute, San Francisco, CA USA; ^4^ Brigham and Women’s Hospital, Harvard Medical School, Boston, MA USA; ^5^ Department of Psychiatry, Neurology, and Epidemiology and Biostatistics University of California San Francisco School of Medicine, San Francisco, CA USA

## Abstract

**Background:**

Increasing evidence has linked sleep quality and sleep apnea to poorer brain health, yet the association between sleep macro‐architecture, nocturnal hypoxemia and Alzheimer’s Disease (AD)‐related patterns on neuroimaging remains less known, especially across older adults from diverse ethnoracial groups.

**Method:**

The recently completed Health and Aging Brain Study‐Health Disparities (HABS‐HD)‐Dormir Study recruited community‐dwelling non‐Hispanic White (NHW), Hispanic, and Black participants who underwent an in‐home sleep apnea assessment (WatchPAT, Itamar, IS) and brain magnetic resonance imaging (MRI) to evaluate sleep and AD‐related MRI biomarkers. Sleep stages were estimated using a validated proprietary algorithm. Our primary outcomes are AD‐signature cortical thickness (in individual regions of interests, including entorhinal cortex, fusiform gyrus, inferior temporal gyrus, and middle temporal gyrus, N = 636) and white matter hyperintensities (WMH) volume [log(WMH+1), normalized by intracranial volume, and categorized into tertiles, N = 842]. We applied multivariable linear or ordinal regression models adjusting for age, sex, ethnicity, education, body mass index, cognitive status, smoking, alcohol consumption, and MRI scanner.

**Result:**

A total of 842 elderly participants [34% male; 42% NHW, 33% Hispanic and 25% Black; age 66.18.6 years] were included in the final analysis. Greater light sleep percentage and longer REM sleep latency were independently associated with thinner cortex in AD‐signature regions: standardized *β*_light sleep percentage_ per 1‐SD increase = ‐0.12 [95% confidence interval (95%CI), ‐0.19 to ‐0.05, false discovery rate (FDR)‐adjusted *p* = 0.007], *β*_REM sleep latency_ per 1‐SD increase = ‐0.14 (95%CI, ‐0.21 to ‐0.07, *p* <0.001); while inverse pattern was observed for deep sleep percentage: *β*_deep sleep percentage_ per 1‐SD increase = 0.12 (95%CI, 0.05 to 0.19, *p* = 0.006) (Figure 1). Higher AHI in REM sleep and mean oxygen saturation<94% (the median value of study sample) were independently associated with greater WMH volume: odds ratio_AHI in REM sleep_ per 1‐SD increase = 1.18 (95%CI, 1.02 to 1.36, *p* = 0.048), odds ratio_mean oxygen saturation<94%_ per 1‐SD increase = 1.38 (95%CI, 1.04 to 1.83, *p* = 0.049) (Figure 2). There were no ethnoracial interactions for these associations.

**Conclusion:**

Light/deep sleep percentage, longer REM sleep latency, and nocturnal hypoxemia were associated with AD‐related MRI patterns.